# Cytoplasmic Streaming in *Neurospora*: Disperse the Plug To Increase the Flow?

**DOI:** 10.1371/journal.pgen.1000526

**Published:** 2009-06-19

**Authors:** Michael Plamann

**Affiliations:** School of Biological Sciences, University of Missouri–Kansas City, Kansas City, Missouri, United States of America; Washington University School of Medicine, United States of America

Filamentous fungi grow as extending and branching tubular cells (hyphae) that generate radially symmetric colonies. As colonies expand, hyphal tips at the periphery avoid each other to allow maximum coverage of the medium, while hyphal tips at the colony center actively fuse to generate an interconnected network of hyphae that allows the bulk movement of cytoplasm toward the colony edge [1]. Hyphal growth is a wonderful adaptation for efficient penetration and acquisition of nutrients from live or dead organisms, especially plants. Unlike multicellular eukaryotes that exist as collections of distinct cells, the syncytial mode of growth places filamentous fungi at serious risk of “bleeding” to death if there is a break in the cell wall. To avoid this fate, fungal hyphae have cross-walls or septa that are perforated to allow the flow of cytoplasm, but can be plugged in response to cellular wounding [2]–[4]. The septal “plug” used by filamentous ascomycete fungi is known as the Woronin body, and it is typically tethered to the rim of the septal pore [4]–[8].

**Figure 1:**
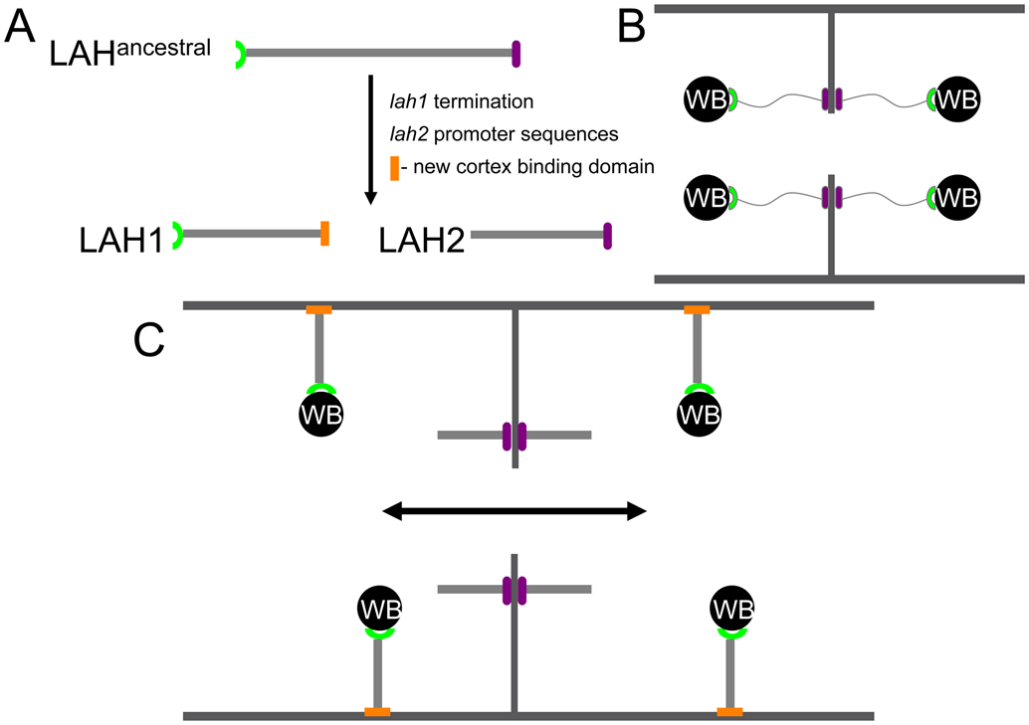
A dispersed septal plug (Woronin body [WB]) may facilitate rapid cytoplasmic streaming and hyphal growth in *N. crassa*. (A) Legend illustrates the ancestral form of Leashin found in most filamentous ascomycetes and the split versions (LAH-1 and LAH-2) found in *N. crassa*. (B) Model for localization of Woronin bodies in the majority of filamentous ascomycetes. Note that LAH connects Woronin bodies to the septal pore rim. In these ascomycetes, cytoplasmic streaming occurs at slow rates, and hyphal diameters and growth rates are reduced relative to *N. crassa*. (C) Model for localization of Woronin bodies in the filamentous ascomycetes *N. crassa*. Note that LAH-1 localizes the Woronin body to the cell cortex and not the septal pore rim; however, LAH-2, lacking the large Woronin body complex, does localize to the septal pore rim. The double-headed arrow indicates the ability of cytoplasm to flow through septal pores in an unimpeded manner. Ng et al. suggest that this modification of LAH tethering protein structure and function may have been a determining event in allowing *N. crassa* to evolve extreme rates of cytoplasmic streaming and hyphal growth. This figure was adapted from Figure 7 of [10].

In the filamentous fungus *Neurospora crassa*, cytoplasmic streaming can occur at incredible rates, up to 60 µm/sec [9]. The typical “trap-door” arrangement of the Woronin body seen in most ascomycetes might be expected to impede rapid flow of large organelles such as nuclei through septa. However, in this issue of *PLoS Genetics*, Seng Kah Ng et al. describe a curious modification of the tethering mechanism for the Woronin body in *N. crassa* [10]. Ng et al. describe the identification of *leashin* (*lah*), a fungal-specific gene that encodes an organellar tether for Woronin body inheritance and cell cortex association. In most of the filamentous ascomycetes, the *lah* gene encodes a single protein; however, in *N. crassa* and the closely related fungus *Sordaria fimicola*, *lah* has been split into two genes, *lah-1* and *lah-2*. The authors show that in *N. crassa*, LAH-1 links Woronin bodies with the cell cortex and not the septal pore. Mutations eliminating LAH-1 function result in loss of Woronin body inheritance and greatly increased “bleeding” of hyphae. LAH-2 was shown to localize to the hyphal apex and the septal pore rim and to play an important role in colony development. These findings have led the authors to propose a model (Figure 1) where the rapid growth rate of *N. crassa* hyphae (>1 µm/sec) is dependent on the extremely high rates of cytoplasmic streaming through septal pores. In fungi with Woronin bodies tethered directly to septal pores, the free flow of cytoplasm and large organelles would be reduced; however, the tethering of Woronin bodies to the cell cortex in *N. crassa* keeps the septal pores clear and allows unrestricted cytoplasmic streaming. As a test of their model, the authors fused the *lah-1* and *lah-2* genes to mimic the *lah* gene organization seen with other filamentous ascomycetes. Interestingly, the strain containing the *lah-1*/*lah-2* gene fusion showed localization of Woronin bodies to both sides of septal pores as in other filamentous ascomycetes, and the fusion protein appeared functional as protoplasmic “bleeding” of damaged hyphae was significantly reduced. Consistent with the authors' prediction that such an arrangement would restrict cytoplasmic streaming, the *lah-1*/*lah-2* strain grew at one-third the rate of wild type.

The Woronin body protein HEX, the Woronin sorting complex protein WSC, and the Leashin protein(s) are found only within the filamentous ascomycetes [2]. The filamentous basidiomycete fungi represent an immense group that have a completely different septum architecture [2],[3]. In some of the filamentous basidiomycetes, there are reports of cytoplasmic flow rates of up to 70 µm/sec [11]. It will be of great interest to see if future work finds that the filamentous basidiomycetes with fast cytoplasmic streaming have also dispersed the “plug” to ensure free flow of materials through septal pores, allowing rapid hyphal and colony growth.
